# Detection to Hospital Door: Gender Differences of Patients With Acute Stroke Symptoms

**DOI:** 10.3389/fneur.2022.833933

**Published:** 2022-04-07

**Authors:** Silke Walter, Daniel Phillips, Brittany Wells, Robert Moon, Thomas Bertsch, Iris Q. Grunwald, Klaus Fassbender

**Affiliations:** ^1^Neurology, Saarland University, Homburg, Germany; ^2^East of England Ambulance Service NHS Trust, Melbourn, United Kingdom; ^3^Institute of Clinical Chemistry, Laboratory Medicine and Transfusion Medicine, Nuremberg General Hospital, Paracelsus Medical University, Nuremberg, Germany; ^4^Division of Imaging Science and Technology, School of Medicine, University of Dundee, Dundee, United Kingdom

**Keywords:** prehospital, acute stroke, management, women, gender inequity

## Abstract

Although prehospital stroke management is challenging, it is a crucial part of the acute stroke chain to enable equal access to highly specialised stroke care. It involves a critical understanding of players usually not specialized in acute stroke treatments. There is contradictory information about gender inequity in prehospital stroke detection, dispatch, and delivery to hospital stroke centers. The aim of this narrative review is to summarize the knowledge of gender differences in the first three stages of acute stroke management. Information on the detection of acute stroke symptoms by patients, their relatives, and bystanders is discussed. Women seem to have a better overall knowledge about stroke, although general understanding needs to be improved. However, older age and different social situations of women could be identified as reasons for reduced and delayed help-seeking. Dispatch and delivery lie within the responsibility of the emergency medical service. Differences in clinical presentation with symptoms mainly affecting general conditions could be identified as a crucial challenge leading to gender inequity in these stages. Improvement of stroke education has to be applied to tackle this inequal management. However, specifically designed projects and analyses are needed to understand more details of sex differences in prehospital stroke management, which is a necessary first step for the potential development of substantially improving strategies.

## Introduction

Acute stroke care and access to timely treatment strongly depend on efficiently organized prehospital management. The stroke chain of survival with its first 3 “Ds”, detection, dispatch, and delivery, based in the prehospital setting emphasizes the importance of optimal pathways before the patients arrive at the hospital (1). There is contradictory information about gender inequity in acute stroke treatment. A German nationwide cohort analysis with >1 million patients identified a higher probability of men receiving stroke unit treatment (OR, 1.11; 95% CI, 1.09–1.12) with a lower in-hospital mortality (OR, 0.91; 95% CI, 0.89–0.93) compared to women (2). However, intravenous thrombolysis (IVT) treatment numbers were similar in this study and more women received endovascular treatment (EVT). A Swedish analysis confirmed significantly lower numbers of women receiving stroke unit care in their cohort (3). In contrast, a retrospective analysis of patients with acute ischemic stroke (AIS) arriving at hospital within 2 h after symptom onset from the American Get-With-The-Guidelines-Stroke registry identified female sex as a risk factor for not receiving IVT (4). It is unclear whether these differences are caused by an already existing gender inequity in the very first prehospital stages of acute stroke care in different regions. This review gives insights into available knowledge on gender differences from symptom onset until arrival at the hospital emergency department and discusses available information on detection, dispatch, and delivery of the acute stroke management cascade.

## Detection of Acute Stroke Symptoms

Identification of acute symptoms of stroke can be challenging even for specialists. However, this is the first crucial step in gaining access to modern stroke treatment, especially to recanalizing therapies with a time-limited treatment window for the best outcome. Most of the current literature describes a better recognition and identification of acute stroke symptoms by women, but there are also publications emphasizing their lack of stroke understanding.

### Differences in Stroke Knowledge

A meta-analysis of 22 studies, of which 20 were cross-sectional and 2 pretest–posttest design surveys, mainly conducted in the USA and Canada, identified a greater knowledge of stroke symptoms and related risk factors in women compared to men (5). In a Spanish randomized study, in which knowledge about stroke symptoms and risk factors was collected with structured face-to-face questionnaires, no sex difference could be detected in general knowledge about stroke, but women showed a higher understanding of risk factors. However, they were less likely to call an ambulance (6). In contrast, in a cross-sectional Chinese study, in which men and women with stroke and hypertension were questioned regarding their stroke knowledge and behavior, men demonstrated a better knowledge about stroke but had a worse pre-stroke health behavior than women (7). An American stroke survey performed with a limited number of stroke survivors detected women as significantly more likely compared to men to identify all the five traditional warning signs of stroke and subsequently take the correct action by calling the Emergency Medical Service [EMS; (8)]. In 4 Canadian cross-sectional surveys, in which public awareness campaigns including information about stroke preceded a stroke-knowledge questionnaire about face, arm, speech symptoms, a clear association of limited knowledge with male sex was found [Odds ratio 0.68; 95% CI: 0.53, 0.86; (9)].

In addition, the overall perception of stroke knowledge in women is poor. A survey of 1,024 women contacted by randomly selected telephone numbers in the USA in 2003 identified that only one-fourth of all women felt well informed about stroke and stroke risk and just below a quarter reported to be very concerned about the disease. Standing out was that younger women aged 25–34 years had the highest rate of nescience (37%). Hispanic or Black ethnicity was associated with less knowledge, but correct identification of acute stroke symptoms was low for all age groups and independent of racial backgrounds (10).

The above examples of available evidence (summarized in Table 1) emphasize that in many countries women are not generally underprivileged in their knowledge about stroke and understanding of the necessary actions to take. But, knowledge in general still needs improvement and differs not only between the countries but also between different ethnic groups. Both latter stress the need for tailored regional educational programmes involving women of all ethnic groups. A one-fit-all approach will not reach the aim.

**Table 1:** Studies addressing gender in prehospital stroke management.

**Reference**	**Location**	**Study design**	**Participants n**	**Results forwomen**
**Detection**				
Bártlová et al. (11)	Czech Republic	Cohort study	1,004	Women show more interest in stroke information
Ferris et al. (10)	USA	Telephone surveywith randomly selected women	1,024	Only 25% women feel well informed
Focht et al. (8)	USA	Cohort survey	71	Women know more stroke signs and more likely to take correct actions
Li et al. (7)	China	Cross-sectional survey	272 men and 118 women	Women less knowledge about men, but better pre-stroke health behavior
Morgenstern et al. (12)	USA	RCT	573	Girls and boys show equal knowledge after stroke education
Mueller-Nordhorn et al. (13)	Germany	RCT	75,720	Women show reduced prehospital times after posted stroke information
Ramírez-Moreno et al. (6)	Spain	RCT	2,409	Women show higher understanding of risk factors, but EMS alerted less often, no difference in general stroke knowledge
Rioux et al. (9)	Canada	Cross-sectional survey	2,451	Women show better knowledge after awareness campaign
Stroebel et al. (5)	USA, Canada	Metaanalysis	20 cross-sectional, 2 pre-posttest surveys	Women show greater knowledge about stroke and risk factors
Umar et al. (14)	USA	Cohort survey	608	No difference in stroke knowledge of girls and boys
**Dispatch**				
Barr et al. (15)	Australia	Cohort study	150	Fewer women recognized the importance of immediate transfer to hospital
Buck et al. (16)	USA	Cohort study	871	More women than men were misidentified by EMS dispatcher
Mainz et al. (17)	Denmark	Cohort study	5,356	Women living alone have longer total prehospital time delay
Mochari-Greenberger et al. (18)	USA	GWTG registry	398,798	Hispanic, Asian, Black women less likely to use the EMS
Springer and Labovitz (19)	USA	Cohort study	1,940	Women more often found down, leads to admission delay
**Delivery**				
Govindarajan et al. (20)	USA	Registry cohort	3,787	Fewer women are correctly diagnosed by EMS
Hsieh et al. (51)	Taiwan	Cohort study	928	More men are pre-notified to hospital
Leung et al. (21)	Hong-Kong	Cohort study	298	More men are pre-notified to hospital, pre-notification associated with shorter treatment times
Lin et al. (22)	USA	GWTG registry	371,998	Fewer women are transferred with pre-notification
Madsen et al. (23)	USA	Cohort study	1,991	Females living alone are at higher risk to have delayed hospital arrival combreaked to men
Mould-Millman et al. (24)	USA	Cohort study	548	Women have lower sensitivity to be correctly diagnosed at the emergency site

Another possibility to increase stroke knowledge is to address pupils rather than only adults and to integrate medical education into school programmes. There is only limited information about the potential benefits of an early school education about stroke and stroke symptoms. However, there seems to be no gender difference. In an 11-question multiple-choice stroke awareness survey with >600 American High school pupils, no difference in stroke symptom knowledge between girls and boys was detected (14). A randomized controlled, multiethnic school-based intervention study called “Kids Identifying and Defeating Stroke” (KIDS), which was started in middle schools enrolling 8,827 pupils in Texas, USA investigated whether a structured stroke education campaign for their year 6 to year 8 pupils could improve stroke knowledge and necessary emergency actions. The programme was performed as four 1-h classes each year, taught by health teachers and neurologists, and included a homework assignment involving parents. A significant increase in stroke knowledge and correct reaction to witnessed symptoms of students' in the KIDS group compared with controls could be observed. The result did not show any gender differences (12). No information could be found from other countries. Also, it stays unclear whether school educational programmes can lead to a longer-lasting increase in the overall understanding of stroke symptoms, and emergency actions needed. This requires further investigation.

### Educational Effect of Public Awareness Campaigns

Public awareness campaigns, comparable to advertisements promoting products or services, are general means that use mass media and new media to transfer information.

It is very well described that most public awareness campaigns can improve stroke knowledge only for a short amount of time, usually lasting between 3 and 6 months (25). Interestingly, it seems that there is a gender difference in susceptibility to such campaigns. A randomized study analyzing the effect of a posted information letter about stroke symptoms showed that the outcome of reduction of prehospital times was only significant for women (13). This could be explained by the finding from a cohort analysis in the Czech Republic, where a sample of 1,004 people were interrogated about their stroke knowledge. Women showed significantly higher interest in the information than men (11). Nevertheless, it stays unclear whether permanently repeated public awareness campaigns in the spirit of “Groundhog Day” could lead to a more solid understanding of stroke of all genders and ethnicities.

## Dispatch: Involvement of EMS

Early activation and dispatch of EMS is a vital element in the acute stroke chain of survival (26). The use of EMS is associated with shorter times to treatment (27); however the existing literature evidences a large degree of variation in how quickly patients alert the EMS and the subsequent dispatch of a medical resource. Prehospital delays can range from 20 min to >150 h based on a number of factors, including the patient's awareness of the severity of their symptoms with data suggesting that female patients with stroke experience an increased delay time in calling for help (15).

There is a paucity of contemporary evidence surrounding sex differences in activation and dispatch of EMS; however there is a recognized inequity in emergency care access in women with stroke, which may be a result of delay in EMS dispatch (28). In a study of 5,515 patients with stroke in the Netherlands, symptom onset to door time was found to be on average 27 min longer in women than in men (29). This finding can be caused by multiple different underlying reasons, of which some are discussed below and are summarized in Table 1.

### Willingness to Call

Existing literature presents an inconsistent picture of sex differences in the activation of EMS. Ramirez-Moreno et al. (6) studied responses from 2,409 participants aged 18 and over, who were surveyed through face-to-face interviews consisting of open-ended questions about the respondent's hypothetical answer to presenting or witnessing signs of a stroke and upon suspecting stroke or transient ischemic attack (TIA) in a family member or themselves. An appropriate response to a suspected stroke was indicated by 83.4% of men compared to only 77.5% of women. These findings are corroborated by further research which suggest that women are more likely to delay accessing emergency care than men, with some literature suggesting that the odds of being admitted to hospital within 3 h of symptom onset was 10% lower for women than men (30). Possible reasons for the disparity in the response to stroke symptoms between men and women may include a difference in perceived severity of the symptoms with women underestimating the urgency of the situation (6). But there are also intrasexual differences. Data analyzed from 398,798 American Get-with-the-guideline stroke registry patients, identified Hispanic or Asian or Black women compared to White women as less likely to use the EMS when stroke symptoms occurred [aOR, 95%CI: Black: 0.87, 0.84–0.91; Hispanic: 0.71, 0.67–0.74; Asian: 0.71, 0.67–0.76; (18)].

### Reasons for Delayed Calling

Delayed calling for help might have multiple underlying causes. An American questionnaire study assessing attitudes toward response to stroke onset found that women, who arrived at hospital over 3 h after symptom onset, cited reasons such as not wanting to trouble others, opting to see if their symptoms might resolve, hiding their symptoms from others, and trying to continue with their normal actives for a delay in seeking emergency care (31). This finding demonstrates some plausible reasons for the delay in EMS dispatch, and therefore hospital arrival, that impacts the eligibility for recanalizing treatments due to arriving outside the treatment window (17).

Whilst the study did not include men and therefore provides no opportunity for comparison with regard to responses, it is recognized that there are sex-specific differences in the way in which medical assistance is summoned following the onset of stroke symptoms, which can result in a delay in dispatch for women (32). In a study of 150 patients with stroke in Australia, it was found that women experienced a delay in symptom onset to presentation to ED that was a mean average of 1.4 h longer than men, with knowledge and recognition of stroke symptoms and not recognizing the importance of their symptoms significantly impacting this delay (15).

### Impact of Difference in Social Situations

If a stroke is witnessed, the activation of EMS is substantially shorter than if symptom onset was unwitnessed (33). It is, therefore, reasonable to suggest that living alone may result in a higher incidence of stroke with an unwitnessed symptom onset and therefore delayed EMS dispatch. Mainz and collaborators published in 2020 (17) the higher proportion of women living alone at the time of their stroke as a likely cause for a longer delay in symptom onset to EMS call. Their study found that this “patient-dependent delay” for women was 19.8 min longer than for men, with living alone being associated with a longer total prehospital time delay. About 50.4% of women lived alone compared to only 31.7% of men.

A retrospective cohort analysis of 1,904 hospital patients with stroke in New York, USA identified that women are significantly more often found with severe stroke symptoms unable to seek help on their own (“found down”) and that this led to all of them arriving 3 h or later after onset (19).

If the onset of stroke symptoms is witnessed, it is most commonly by the patient's partner (34); however more women than men are widowed at the time of stroke and therefore live alone, with a delay in EMS dispatch a plausible consequence of this (35). The caller was the patient themselves in only 3% of cases based on a study of 198 patients transported to the emergency department (ED) by EMS in Melbourne (34), further supporting the hypothesis that women are susceptible to delays in EMS dispatch.

Another reason adding to the gender difference in EMS activation might be that women are older than men at the time of stroke, particularly in Europe, Australia, and South America (36). This results in a higher level of functional limitation prior to their stroke (35, 37), which may impact their physical ability to summon help.

### Role of the Emergency Medical Service Dispatch Center

The identification of stroke symptoms at the point of EMS dispatch is key in optimizing the chain of survival in acute stroke and this remains one of the least investigated elements of the chain (38). It is critical that dispatch centers accurately identify stroke to avoid the assignment of an incorrect “code” and cause subsequent delays in EMS dispatch (39). There are many differences in the organization of EMS worldwide (40); however findings show a stark variation in the proportion of strokes correctly identified at the point of dispatch with successful identification ranging from 45 to 83% (16, 41).

In some countries, the Medical Priority Dispatch System (MPDS) is used for EMS dispatch. This system is based on a structured caller interrogation with additional instructions on what to do until EMS arrival. In a Los Angeles study of 871 patients, 58 were assigned the stroke MPDS code by the dispatcher, but only 45% received a confirmed in-hospital stroke diagnosis (16). A total of 56.2% of female patients with stroke were misidentified in this study. In a review of existing literature, Oostema and collaborators (42) found that despite the use of a stroke screening tool, the recognition of stroke by the dispatch center was inadequate and it is suggested that many subtle stroke presentations may be misidentified (39). A barrier to the identification of stroke symptoms at the point of dispatch is the variety of presenting symptoms that may fall outside of the screening system adopted by the communication center. With women more likely to experience non-traditional stroke symptoms, an inherent risk not only of incorrect call coding at the point of dispatch with subsequent delays in timely EMS care but also of EMS identification of a woman with stroke, decelerating the delivery to the stroke center, has to be considered.

## Delivery to Hospital

There are many reports on differences in clinical presentation between men and women [Table 1; (43)]. This likely not only affects stroke patient's identification at the dispatch center level but also at the scene, influencing the delivery step of the acute stroke chain of survival. Delivery comprises of rapid EMS identification of stroke symptoms, management of the patient on the scene, and timely transportation and prenotification to the hospital, as defined by the American Heart Association (44).

### Gender Differences in Stroke Presentation and EMS Recognition

Women are reported to often present with unusual stroke symptoms such as generalized weakness, fatigue, and mental status change. In an analysis of 461 patients, 52% of women compared to 44% of men presented with a non-traditional stroke symptom (43). This result was primarily driven by the number of women with mental status changes, a symptom caused by many other differential diagnoses (45). Further studies confirmed that non-traditional stroke symptoms were present in 51.8% of women compared to 43.9% of men (46). In addition, disorientation, visual disturbance, dizziness headache, general pain, urinary incontinence, or changes in consciousness are specifically described to be shown in female stroke (47, 48).

These reported differences in the clinical presentation of stroke between genders can therefore be of challenge for the EMS when diagnosing stroke in the prehospital environment with only limited diagnostic tools available. This is supported by multiple studies (49). A recent systematic meta-analysis of 21 observational studies with 6,934 stroke and transient ischemic attack patients identified that 26% of all patients with stroke, who were missed by the EMS, presented with non-FAST symptoms like speech abnormalities, nausea/vomiting, dizziness, changes in mental status, and visual disturbance (41). A study with 3,787 patients transported by EMS found that only 30% of women compared to 35% of men received a correct diagnosis of stroke, with these findings perceived to be based on the non-traditional stroke symptoms displayed more frequently by women (20).

Various measures have been previously discussed to enhance prehospital stroke recognition, including the use of validated stroke scales (50). A retrospective analysis of 548 emergency patients in Atlanta, USA evaluated that paramedics were more likely to positively identify stroke when the Cincinnati Prehospital Stroke Scale (CPSS) was positive. However, sensitivity to be diagnosed at the scene was lower for women than men [odds ratio 0.53, 95%CI 0.17–1.63; (24)].

It is likely that different presentations of women with acute stroke lead to a wrong working diagnosis by the EMS in the prehospital phase, which could impact further stroke management. However, more prospective studies are needed to understand the real impact.

### Gender Differences in EMS On-Scene Time Metrics and Prenotification to Hospital

A large study of nearly 2,000 patients presenting with AIS found that before adjustment, the time of symptom onset to arrival at the hospital *via* the EMS was slightly longer in women (mean 337 min vs. 297 min in men); however, they subsequently found that gender was not associated with a delayed time to arrival when considering age and National Institute Of Health Stroke Severity (NIHSS) score. It was demonstrated that 30% of women in the study (324/1097) lived alone compared to 22% of men (200/894), and that this was a factor in delayed arrival at the hospital, potentially due to the lack of self-recognition of symptoms (23). A study of 5,356 patients identified that 40.5% of women and 44.4% of men arrived at the stroke unit within 3 h of symptom onset, and similarly living alone was deemed to be a contributory factor involved in this finding. About 54.4% (1,256) of women were documented as living alone, leading to an average delay of 20 min longer than their male counterparts (17).

A study from 2016 demonstrated an association not only between pre-notification and faster door to computed tomography (CT) scan in patients presenting within 3 h of symptom onset but also a shorter door-to-needle (DTN) time. This study comprised of 928 patients, of whom 727 received pre-notification to the hospital. There was a significantly higher number of men who were transported with pre-notification (64.5%), and more pre-notified patients had a DTN of <60 min (45.1 vs. 28%) compared to those not pre-notified (51). A similar pattern was shown in a study from Hong Kong, which found that the ratio of men to women receiving pre-notification to hospital for stroke was 1.22:1. Pre-notification was also demonstrated to improve door-to-CT and DTN time (21).

An analysis of the American Get-with-the-Guideline stroke registry of 371,998 enrolled patients with stroke in 1,585 hospitals over 8 years showed a pre-notification rate of 67%. Patients with EMS pre-notification to the hospital were more likely to be younger, white, and male. In the 122,791 patients eligible for pre-notification where none was given, 54.3% were women (22).

These differences in prehospital management of women with acute stroke might be caused by a higher number of misdiagnoses, subsequently leading to lower numbers with accurately initiated stroke alert to the hospital. But clearly, further research is required to understand unbiased differences in prehospital treatment and outcomes.

## Discussion

This review addresses gender differences in the very first stages of acute stroke management, the detection of disease symptoms to the delivery of the suspected stroke patient to the specialized center. It highlights some of the available evidence with the aim to raise awareness and identify a pattern, hinting toward areas of necessary improvement to guarantee gender equity in prehospital acute stroke treatment.

Detection of acute stroke and initiation of the necessary steps, which enable health care specialists to administer up-to-date and high-quality stroke care, is incumbent to the patient and or relatives or bystanders. There is a lot of evidence coming from different countries that women have a better knowledge about acute stroke symptoms, risk factors, and the necessary actions to take than men. However, the overall public level of knowledge about stroke, the number three disease cause for disability is still poor (52, 53). Gender inequity in the detection of stroke seems to show geographical differences, but further systematic evaluations are needed to understand where and what differences exist.

Despite often having a higher and better knowledge about stroke, women seem to feel uncertain about many aspects of the disease. Women's confidence in their own knowledge differs from that of men (54), which needs to be considered and addressed in educational programmes.

The idea to implement health education in standard school programmes would be one possibility to establish gender-independent knowledge transfer in the future. But, to reach girls and young women in countries with relevant sex inequity would need strong collaboration between health services and educational sectors with strong political and governmental support.

It is well described that public campaigns do not lead to a sustainable solution to improve health awareness (1); however it seems that women are more likely and willing to pick up information provided by mass media campaigns (55).

However, equal knowledge and awareness alone do not lead to equal acute stroke care. The available evidence suggests that women arrive at the hospital later than men, indicating an inequity in dispatch and delivery of acute stroke care. Relevant confounders like social disparity with more women living alone at an older age, when the risk of stroke increases, have to be considered. Management of women seeking help at an emergency medical dispatch center does not seem to show any gender-based differences. But many countries use systematized interrogation programmes, like the Medical Priority Dispatch System to identify stroke suspects, which could be prone to errors and disadvantageous to women with stroke, who often present with non-traditional symptoms (43).

There is no information available on whether a criteria-based dispatch, used in many Nordic and European countries (56), which relies on the experience of the telecommunicator, is less prone to misidentify women with suspected strokes. More studies comparing both modes of emergency service dispatch are needed to identify gender-related challenges of different dispatch systems.

The differences in the clinical presentation of women compared to men can make it difficult for paramedics to quickly conclude a stroke working diagnosis at the emergency site. This likely presents the biggest challenge in the delivery of equal care. There is evidence for an inequity in pre-notification of cases to the receiving hospital. To tackle this, paramedic and EMS stroke-specific training have to be considered and addressed. A correlation between high-quality medical education and patient outcome has been demonstrated for nurse care (57, 58), but data for the EMS or, more important, training programme adjustment for prehospital staff is lacking.

To conclude, more structured data and results from specifically designed clinical trials are needed to understand gender inequity in the first stages of acute stroke care and to develop solutions to overcome potential gender disbalances. Identified gender inequity is mostly caused by unawareness of gender-specific aspects of stroke, which are not considered in acute prehospital pathways.

## Author Contributions

SW, BW, RM, and KF contributed to the conception and design of the review. SW, BW, and RM wrote the first draft of the manuscript. All authors contributed to manuscript revision, read, and approved the submitted version.

## Conflict of Interest

The authors declare that the research was conducted in the absence of any commercial or financial relationships that could be construed as a potential conflict of interest.

## Publisher's Note

All claims expressed in this article are solely those of the authors and do not necessarily represent those of their affiliated organizations, or those of the publisher, the editors and the reviewers. Any product that may be evaluated in this article, or claim that may be made by its manufacturer, is not guaranteed or endorsed by the publisher.

## References

[1] Fassbender K Walter S Grunwald IQ Merzou F Mathur S Lesmeister M. Prehospital stroke management in the thrombectomy era. Lancet Neurol. (2020) 19:601–10. 10.1016/S1474-4422(20)30102-2 32562685

[2] Weber R Krogias C Eyding J Bartig D Meves SH Katsanos AH. Age and sex differences in ischemic stroke treatment in a nationwide analysis of 111 million hospitalized cases. Stroke. (2019) 50:3494–502. 10.1161/STROKEAHA.119.026723 31623547

[3] Dahl S Hjalmarsson C Andersson B. Sex differences in risk factors, treatment, and prognosis in acute stroke. Womens Health. (2020) 16:1745506520952039. 10.1177/1745506520952039 32997605 PMC7533936

[4] Messé SR Khatri P Reeves MJ Smith EE Saver JL Bhatt DL. Why are acute ischemic stroke patients not receiving IV tPA? Results from a national registry. Neurology. (2016) 87:1565–74. 10.1212/WNL.0000000000003198 27629092 PMC5067546

[5] Stroebele N Müller-Riemenschneider F Nolte CH Müller-Nordhorn J Bockelbrink A Willich SN. Knowledge of risk factors, and warning signs of stroke: a systematic review from a gender perspective. Int J Stroke. (2011) 6:60–6. 10.1111/j.1747-4949.2010.00540.x 21205242

[6] Ramírez-Moreno JM Alonso-González R Peral-Pacheco D Millán-Núñez MV Aguirre-Sánchez JJ. Knowledge of stroke a study from a sex perspective. BMC Res Notes. (2015) 8:604. 10.1186/s13104-015-1582-1 26499113 PMC4620012

[7] Li ZR Ruan HF Shen LP Zhang XP Wan LH. Gender difference in the association between stroke knowledge and health behavior before the onset of stroke among Chinese hypertensive patients. J Neurosci Nurs. (2021) 53:160–5. 10.1097/JNN.0000000000000599 34116556

[8] Focht KL Gogue AM White BM Ellis C. Gender differences in stroke recognition among stroke survivors. J Neurosci Nurs. (2014) 46:18–22. 10.1097/JNN.0000000000000026 24399163

[9] Rioux B Brissette V Marin FF Lindsay P Keezer MR Poppe AY. The Impact of Stroke Public Awareness Campaigns Differs Between Sociodemographic Groups. Can J Neurol Sci. (2021) 20:1–8. 10.1017/cjn.2021.76 33875043

[10] Ferris A Robertson RM Fabunmi R Mosca L American Heart Association American Stroke Association. American Heart Association and American Stroke Association national survey of stroke risk awareness among women. Circulation. (2005) 111:1321–6. 10.1161/01.CIR.0000157745.46344.A1 15769775

[11] Bártlová S Šedová L Rolantová L Hudáčková A Dolák F Sadílek P. General awareness of stroke in the Czech Republic. Cent Eur J Public Health. (2021) 29:230–5. 10.21101/cejph.a6212 34623124

[12] Morgenstern LB Gonzales NR Maddox KE Brown DL Karim AP Espinosa N. randomized, controlled trial to teach middle school children to recognize stroke and call 911: the kids identifying and defeating stroke project. Stroke. (2007) 38:2972–8. 10.1161/STROKEAHA.107.490078 17885255

[13] Müller-Nordhorn J Wegscheider K Nolte CH Jungehülsing GJ Rossnagel K Reich A. Population-based intervention to reduce prehospital delays in patients with cerebrovascular events. Arch Intern Med. (2009) 169:1484–90. 10.1001/archinternmed.2009.232 19752406

[14] Umar AB Koehler TJ Zhang R Gilbert V Farooq MU Davis AT. Stroke knowledge among middle and high school students. J Int Med Res. (2019) 47:4230–41. 10.1177/0300060519858887 31307252 PMC6753559

[15] Barr J McKinley S O'Brien E Herkes G. Patient recognition of and response to symptoms of TIA or stroke. Neuroepidemiology. (2006) 26:168–75. 10.1159/000091659 16493205

[16] Buck BH Starkman S Eckstein M Kidwell CS Haines J Huang R. Dispatcher recognition of stroke using the National Academy Medical Priority Dispatch System. Stroke. (2009) 40:2027–30. 10.1161/STROKEAHA.108.545574 19390065 PMC2711028

[17] Mainz J Andersen G Valentin JB Gude MF Johnsen SP. Disentangling sex differences in use of reperfusion therapy in patients with acute ischemic stroke. Stroke. (2020) 51:2332–8. 10.1161/STROKEAHA.119.028589 32640943

[18] Mochari-Greenberger H Xian Y Hellkamp AS Schulte PJ Bhatt DL Fonarow GC. Racial/ethnic and sex differences in emergency medical services transport among hospitalized us stroke patients: analysis of the national get with the guidelines-stroke registry. J Am Heart Assoc. (2015) 4:e002099. 10.1161/JAHA.115.002099 26268882 PMC4599467

[19] Springer MV Labovitz DL. The effect of being found with stroke symptoms on predictors of hospital arrival. J Stroke Cerebrovasc Dis. (2018) 27:1363–7. 10.1016/j.jstrokecerebrovasdis.2017.12.024 29428327

[20] Govindarajan P Friedman BT Delgadillo JQ Ghilarducci D Cook LJ Grimes B. Race and sex disparities in prehospital recognition of acute stroke. Acad Emerg Med. (2015) 22:264–72. 10.1111/acem.12595 25728356 PMC4355063

[21] Leung WCY Teo KC Kwok WM Lam LHC Choi OMY Tse MMY. Pre-hospital stroke screening and notification of patients with reperfusion-eligible acute ischaemic stroke using modified Face Arm Speech Time test. Hong Kong Med J. (2020) 26:479–85. 10.12809/hkmj208552 33284132

[22] Lin CB Peterson ED Smith EE Saver JL Liang L Xian Y. Emergency medical service hospital prenotification is associated with improved evaluation and treatment of acute ischemic stroke. Circ Cardiovasc Qual Outcomes. (2012) 5:514–22. 10.1161/CIRCOUTCOMES.112.965210 22787065

[23] Madsen TE Sucharew H Katz B Alwell KA Moomaw CJ Kissela BM. Gender and time to arrival among ischemic stroke patients in the greater cincinnati/Northern Kentucky Stroke Study. J Stroke Cerebrovasc Dis. (2016) 25:504–10. 10.1016/j.jstrokecerebrovasdis.2015.10.026 26617327 PMC4779702

[24] Mould-Millman NK Meese H Alattas I Ido M Yi I Oyewumi T. Accuracy of prehospital identification of stroke in a large stroke belt municipality. Prehosp Emerg Care. (2018) 22:734–42. 10.1080/10903127.2018.1447620 29596006

[25] Fassbender K Grotta JC Walter S Grunwald IQ Ragoschke-Schumm A Saver JL. Mobile stroke units for prehospital thrombolysis, triage, and beyond: benefits and challenges. Lancet Neurol. (2017) 16:227–37. 10.1016/S1474-4422(17)30008-X 28229894

[26] Ashcraft S Wilson SE Nyström KV Dusenbury W Wira CR Burrus TM. Care of the patient with acute ischemic stroke (prehospital and acute phase of care): update to the 2009 comprehensive nursing care scientific statement: a scientific statement from the American Heart Association. Stroke. (2021) 52:e164–78. 10.1161/STR.0000000000000356 33691468

[27] Bray JE Mosley I Bailey M Barger B Bladin C. Stroke public awareness campaigns have increased ambulance dispatches for stroke in Melbourne, Australia. Stroke. (2011) 42:2154–7. 10.1161/STROKEAHA.110.612036 21757668

[28] Powers WJ Rabinstein AA Ackerson T Adeoye OM Bambakidis NC Becker K. Guidelines for the early management of patients with acute ischemic stroke: 2019 update to the 2018 guidelines for the early management of acute ischemic stroke: a guideline for healthcare professionals from the American Heart Association/American Stroke Association. Stroke. (2019) 50:e344–418. 10.1161/STR.0000000000000211 31662037

[29] de Ridder I Dirks M Niessen L Dippel D PRACTISE Investigators. Unequal access to treatment with intravenous alteplase for women with acute ischemic stroke. Stroke. (2013) 44:2610–2. 10.1161/STROKEAHA.113.002263 23887843

[30] Foerch C Misselwitz B Humpich M Steinmetz H Neumann-Haefelin T Sitzer M. Arbeitsgruppe Schlaganfall Hessen. Sex disparity in the access of elderly patients to acute stroke care. Stroke. (2007) 38:2123–6. 10.1161/STROKEAHA.106.478495 17525398

[31] Beal CC. Women's interpretation of and cognitive and behavioral responses to the symptoms of acute ischemic stroke. J Neurosci Nurs. (2014) 46:256–66. 10.1097/JNN.0000000000000083 25188683

[32] Mandelzweig L Goldbourt U Boyko V Tanne D. Perceptual, social, and behavioral factors associated with delays in seeking medical care in patients with symptoms of acute stroke. Stroke. (2006) 37:1248–53. 10.1161/01.STR.0000217200.61167.39 16556885

[33] Rosamond WD Gorton RA Hinn AR Hohenhaus SM Morris DL. Rapid response to stroke symptoms: the Delay in Accessing Stroke Healthcare (DASH) study. Acad Emerg Med. (1998) 5:45–51. 10.1111/j.1553-2712.1998.tb02574.x 9444342

[34] Mosley I Nicol M Donnan G Patrick I Dewey H. Stroke symptoms and the decision to call for an ambulance. Stroke. (2007) 38:361–6. 10.1161/01.STR.0000254528.17405.cc 17204685

[35] Willers C Lekander I Ekstrand E Lilja M Pessah-Rasmussen H Sunnerhagen KS. Sex as predictor for achieved health outcomes and received care in ischemic stroke and intracerebral hemorrhage: a register-based study. Biol Sex Differ. (2018) 9:11. 10.1186/s13293-018-0170-1 29514685 PMC5842547

[36] Carcel C Wang X Sandset EC Delcourt C Arima H Lindley R. Sex differences in treatment and outcome after stroke: Pooled analysis including 19,000 participants. Neurology. (2019) 93:e2170–80. 10.1212/WNL.0000000000008615 31719135

[37] Phan HT Blizzard CL Reeves MJ Thrift AG Cadilhac DA Sturm J. Factors contributing to sex differences in functional outcomes and participation after stroke. Neurology. (2018) 90:e1945–53. 10.1212/WNL.0000000000005602 29703773

[38] Puolakka T Strbian D Harve H Kuisma M Lindsberg PJ. Prehospital phase of the stroke chain of survival: a prospective observational study. J Am Heart Assoc. (2016) 5:e002808. 10.1161/JAHA.115.002808 27139735 PMC4889170

[39] Ramanujam P Guluma KZ Castillo EM Chacon M Jensen MB Patel E. Accuracy of stroke recognition by emergency medical dispatchers and paramedics–San Diego experience. Prehosp Emerg Care. (2008) 12:307–13. 10.1080/10903120802099526 18584497

[40] Sikka N Margolis G. Understanding diversity among prehospital care delivery systems around the world. Emerg Med Clin North Am. (2005) 23:99–114. 10.1016/j.emc.2004.09.007 15663976

[41] Jones SP Bray JE Gibson JM McClelland G Miller C Price CI. Characteristics of patients who had a stroke not initially identified during emergency prehospital assessment: a systematic review. Emerg Med J. (2021) 38:387–93. 10.1136/emermed-2020-209607 33608393 PMC8077214

[42] Oostema JA Carle T Talia N Reeves M. Dispatcher Stroke Recognition Using a Stroke Screening Tool: A Systematic Review. Cerebrovasc Dis. (2016) 42:370–7. 10.1159/000447459 27348228

[43] Bushnell C Howard VJ Lisabeth L Caso V Gall S Kleindorfer D. Sex differences in the evaluation and treatment of acute ischaemic stroke. Lancet Neurol. (2018) 17:641–50. 10.1016/S1474-4422(18)30201-1 29914709

[44] Jauch EC Cucchiara B Adeoye O Meurer W Brice J Chan YY. Part 11: adult stroke: 2010 American Heart Association Guidelines for Cardiopulmonary Resuscitation and Emergency Cardiovascular Care. Circulation. (2010) 122:S818–28. 10.1161/CIRCULATIONAHA.110.971044 20956227

[45] Patti L Gupta M. Change In Mental Status. 2021 Aug 11. In: StatPearls. Treasure Island (FL): StatPearls Publishing (2021). 28723002

[46] Branyan TE Sohrabji F. Sex differences in stroke co-morbidities. Exp Neurol. (2020) 332:113384. 10.1016/j.expneurol.2020.113384 32585156 PMC7418167

[47] Lisabeth LD Brown DL Hughes R Majersik JJ Morgenstern LB. Acute stroke symptoms: comparing women and men. Stroke. (2009) 40:2031–6. 10.1161/STROKEAHA.109.546812 19228858

[48] Girijala RL Sohrabji F Bush RL. Sex differences in stroke: review of current knowledge and evidence. Vasc Med. (2017) 22:135–45. 10.1177/1358863X16668263 27815349

[49] Brandler ES Sharma M McCullough F Ben-Eli D Kaufman B Khandelwal P. Prehospital stroke identification: factors associated with diagnostic accuracy. J Stroke Cerebrovasc Dis. (2015) 24:2161–6. 10.1016/j.jstrokecerebrovasdis.2015.06.004 26159643

[50] Fassbender K Balucani C Walter S Levine SR Haass A Grotta J. Streamlining of prehospital stroke management: the golden hour. Lancet Neurol. (2013) 12:585–96. 10.1016/S1474-4422(13)70100-5 23684084

[51] Hsieh MJ Tang SC Chiang WC Tsai LK Jeng JS Ma MH. Effect of prehospital notification on acute stroke care: a multicenter study. Scand J Trauma Resusc Emerg Med. (2016) 24:57. 10.1186/s13049-016-0251-2 27121501 PMC4847216

[52] GBD 2019 Stroke Collaborators. Global, regional, and national burden of stroke and its risk factors, 1990-2019: a systematic analysis for the Global Burden of Disease Study 2019. Lancet Neurol. (2021) 20:795–820. 10.1016/S1474-4422(21)00252-0 34487721 PMC8443449

[53] Melak AD Wondimsigegn D Kifle ZD. Knowledge, prevention practice and associated factors of stroke among hypertensive and diabetic patients - a systematic review. Risk Manag Healthc Policy. (2021) 14:3295–310. 10.2147/RMHP.S324960 34408515 PMC8364969

[54] Bleidorn W Arslan RC Denissen JJ Rentfrow PJ Gebauer JE Potter J. Age and gender differences in self-esteem-A cross-cultural window. J Pers Soc Psychol. (2016) 111:396–410. 10.1037/pspp0000078 26692356

[55] Hodgson C Lindsay P Rubini F. Can mass media influence emergency department visits for stroke? Stroke. (2007) 38:2115–22. 10.1161/STROKEAHA.107.484071 17540967

[56] Bohm K Kurland L. The accuracy of medical dispatch-a systematic review. Scand J Trauma Resusc Emerg Med. (2018) 26:94. 10.1186/s13049-018-0528-8 30413213 PMC6230269

[57] Aiken LH Sloane DM Bruyneel L Van den Heede K Griffiths P Busse R. Nurse staffing and education and hospital mortality in nine European countries: a retrospective observational study. Lancet. (2014) 3830:1824–30. 10.1016/S0140-6736(13)62631-8 24581683 PMC4035380

[58] Middleton S Grimley R Alexandrov AW. Triage, treatment, and transfer: evidence-based clinical practice recommendations and models of nursing care for the first 72 hours of admission to hospital for acute stroke. Stroke. (2015) 46:e18–25. 10.1161/STROKEAHA.114.006139 25563641

